# A Review of the Common Models Used in Mechanistic Studies on Demineralization-Remineralization for Cariology Research

**DOI:** 10.3390/dj5020020

**Published:** 2017-06-18

**Authors:** Ollie Yiru Yu, Irene Shuping Zhao, May Lei Mei, Edward Chin-Man Lo, Chun-Hung Chu

**Affiliations:** Faculty of Dentistry, The University of Hong Kong, Hong Kong SAR 999077, China; yuyiru@hku.hk (O.Y.Y.); irenezhao110@gmail.com (I.S.Z.); mei1123@hku.hk (M.L.M.); hrdplcm@hkucc.hku.hk (E.C.-M.L.)

**Keywords:** demineralization, remineralization, fluoride, caries, review

## Abstract

Mechanistic studies on demineralization-remineralization play a critical role in investigating caries pathogenicity, testing effects of new caries prevention methods, and developing new caries-preventing products. Simulating the cariogenic challenges in the mouth, various demineralization-remineralization models have been used for cariology research. This review aimed to provide an overview of the common mechanistic studies on demineralization-remineralization for cariology research in recent literature. Most mechanistic studies were in vitro studies (*n* = 294, 84%) among the 350 cariology studies indexed in the Web of Science from 2014 to 2016. Among these in vitro studies, most studies (257/294, 87%) used chemical models that could be classified as simple mineralization models (159/257, 62%) or pH-cycling models (98/257, 38%). In vitro studies consumed less expense and time than in vivo studies. Furthermore, in vitro conditions were easier to control. However, they could hardly imitate the complex structures of oral cavities, the microbiological effect of oral biofilm, and the hydrodynamic instability of saliva. The advantages of chemical models included simplicity of the study, low cost, efficiency (time saving), reproducibility, and stability of experiments. However, the “caries” generated were not biological. Moreover, the chemical models were generally basic and could not mimic a carious lesion in the complex oral environment.

## 1. Introduction

Dental caries is the localized destruction of dental hard tissues by acidic byproducts from bacterial fermentation of dietary carbohydrates [1]. It forms through a complex interaction over time between acid-producing bacteria and fermentable carbohydrates, and has many host factors including teeth and saliva. Despite many years of research and the availability of novel anti-caries products, dental caries is still one of the most prevalent chronic diseases affecting many people worldwide [2]. In the past decades, thousands of in vivo and in vitro studies on cariology have been published. Many of them are mechanistic studies that play an important role in cariology research in investigating caries pathogenicity, testing effects of new caries prevention methods (i.e., devices and drugs), and developing new caries-preventing products [2]. A mechanistic study can be defined as an experiment or test to analyze the biological and/or chemical events responsible for, or associated with, an observed effect (outcome). Mechanistic studies on demineralization-remineralization explore the molecular and physiological mechanisms by which substances exert their effects on teeth. The purpose of this review is to provide an overview of the common mechanistic studies on demineralization-remineralization in recent literature on cariology research.

## 2. Types of Mechanistic Studies in Recent Publications

**Figure 1:**
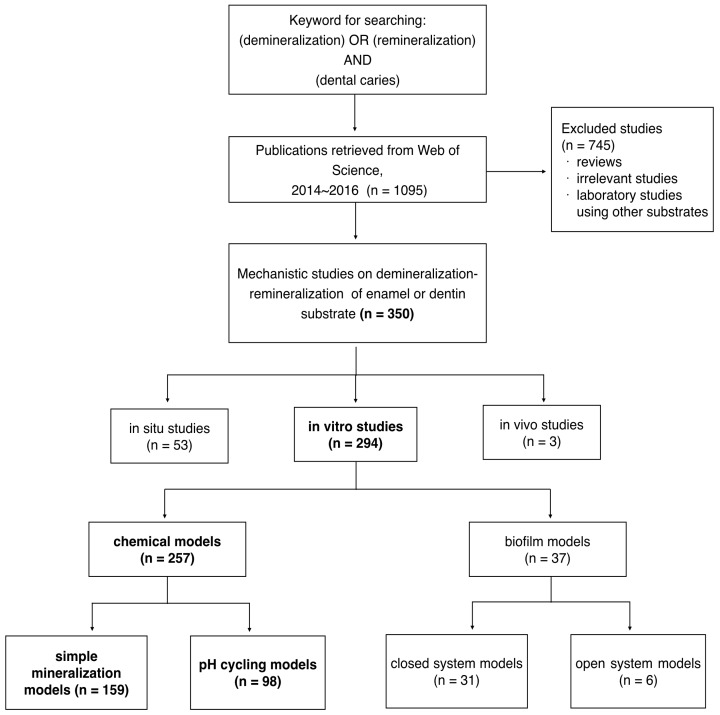
Mechanistic studies on demineralization-remineralization for cariology research published in Web of Science 2014–2016.

A publication search was conducted using Web of Science, which is the first comprehensive scientific citation indexing database used by academics and researchers [3]. Web of Science is in a dominant position in the citation of academic references [4]. All the literature in the database has been published in journals with impact factors. A search using the keywords (demineralization OR remineralization) AND (dental caries) found 350 mechanistic studies from 2014 to 2016 using demineralization-remineralization models of enamel or dentin substrate (Figure 1). These mechanistic studies can be classified into three categories: in vitro studies (294 studies, 294/350, 84%); in situ studies including natural caries studies (53 studies, 53/350, 15%); and in vivo studies (3/350, 1%). Among the 294 in vitro studies, 257 used chemical models and 37 used biofilm models for the study of demineralization-remineralization of teeth (Figure 1). Since one common database with three main keywords were used, this review is not an exhaustive search and is not a systematic review. However, the results of the search provided an outline of common types of mechanistic studies on demineralization-remineralization for cariology research in recent literature.

### 2.1. In Situ Studies

A small number (53/350, 15%) of the recent publications were in situ studies. In situ studies are proposed to make a balance of merits and limitations between in vitro and in vivo studies. Some in situ studies closely mimic the natural environment and process of dental caries formation in vivo. In situ studies examine dental caries exactly in place where they occur. They provide essential information about the physical-chemical characteristics of the dental ecosystem. In situ studies generally have four elements, which are human or animal tooth substrate, cariogenic dental biofilm, regular carbohydrate challenge, and valid reaction time according to the caries generation process and experimental design [5]. They can be used to study the interaction of anti-caries agents and environments. 

Recent in situ studies have explored the process of demineralization and remineralization of enamel and/or dentine, or investigated the effectiveness of caries prevention agents or devices. The most common method of these studies was the use of palatal devices as the carrier of dental substrates. Some studies used extracted teeth with natural caries as specimens for evaluation. Unlike artificial caries, the formation of natural caries could not be controlled. Moreover, the position, type, depth, and other factors of the caries in different teeth were not similar. Therefore, it was difficult to use natural caries models to evaluate the effects of anti-caries agents because the parameters from different caries are incomparable. 

The duration of the in situ studies was normally less than two months. The study designs, to some extent, were similar to in vivo studies. The substrates could be analyzed quantitatively to improve the sensitivity and validity of the methodology. Nevertheless, no actual carious deficiencies would be formed due to the short experimental period. The results, therefore, could not be comparable to those found in clinical situations. In situ studies normally recruited a small number of subjects. The results might not be representative because of individual variation. Furthermore, the results of the experiments highly relied on the compliance of the subjects [6].

### 2.2. In Vivo Studies

In vivo studies allow for the evaluation of the overall effects of an intervention on dental caries in the oral cavity. Rodents are animals commonly used in in vivo studies to study dental caries [7]. Animal studies provides higher scientific control and easier adaptability to calibration than in situ studies. Caries lesions induced in animal studies can greatly mimic the natural caries formation process [7]. Most aspects of caries formation, including such roles of diet, microbiological elements, tooth composition, and de-/remineralization processes, as well as their interactions can be simulated [8]. Smooth surface, fissure, and cervical caries can all be simulated with similar histological features to human caries. Despite the advantages mentioned above, animal studies do not predict clinical outcomes when it comes to anti-caries effect assessments for intervention such as fluoride application. Some animal studies exhibited different results from in vitro and in situ experiments [6]. Last but not least, the uncontrollable fluctuating oral environment and ethical problems associated with in vivo studies in humans made it difficult to study the mechanism of demineralization-remineralization of dental hard tissue [9].

Only a nonsignificant number (3/350, 1%) of the recent studies in this search were in vivo studies. These three studies were animal studies using rats as the subjects to test new anti-caries methods. The International Association Against Painful Experiments on Animals proclaimed that direct extrapolation from animals to humans was frequently invalid. This can be one of the main reasons that animal studies were uncommon in the recent studies [10].

### 2.3. In Vitro Studies

Researchers often use in vitro studies to study the demineralization-remineralization process in cariology research. In vitro studies simulate the oral environment from different perspectives and offer more controllable conditions than a natural environment. The designs of in vitro studies are often simple but can be complex according to the purposes of investigation. Even with a complex design, authors of in vitro studies find it almost impossible to mimic the complicated process of natural caries development. Hence, the design can be regarded as a compromise on the reality of the in vivo ecosystem. Nevertheless, researchers can still obtain meaningful and useful results. The simplification of the in vitro study environment provides an alternative choice to conducting reproducible experiments in a controllable and simplified way [11]. 

The search suggested that in vitro studies were the most common mechanistic studies of cariology in recent years (294/350, 84%). In vitro studies consumed the least expense and time among all the laboratory studies. Furthermore, the conditions were simple and easy to control to meet the research requirements [6]. However, they can hardly imitate the complex structure of the oral cavity, the microbiological effect of oral biofilm, and the hydrodynamic instability of saliva. Different models were used in the in vitro studies. Each model had its own study design. The results can vary significantly because the conditions of caries formation and the characteristics of carious lesion can be very different from natural caries [10]. Moreover, the morphology and mineral loss created by the carious lesions vary significantly in different demineralization-remineralization models [12].

## 3. Demineralization-Remineralization Models Used for In Vitro Studies

The demineralization-remineralization models used for in vitro studies can be divided into biofilm models and chemical models. The biofilm models can be further divided into closed system models and open system models. The chemical models can be further divided into simple mineralization models and pH-cycling models. 

### 3.1. Biofilm Models

Cariogenic bacteria were one of the key factors that influence the characteristics of carious lesions [13]. Some of the recent studies (37/294, 13%) used biofilm models with cariogenic bacteria. Nineteen out of 37 studies employed a pure culture system, which used a single strain of bacteria to provide cariogenic challenge. *Streptococcus mutans* was commonly used (18/19, 95%) as a single species within a pure culture system [14]. The structure and the composition of the mono-species biofilm is consistent. The bacteria cell growth and accumulation rate, as well as the physiological properties of the biofilm, can be accurately investigated. It also allows for the analysis of differences between or among different oral pathogenic species. However, interactions of bacteria, such as competition, cross-feeding, or succession of the colony are not considered [13]. Cariogenic consortia biofilms with a combination of two or more cariogenic bacteria were also used in seven studies. All of them were the co-cultivation of *streptococcus mutans* and *lactobacillus*, *bifidobacteria*, or *actinomyces.* The defined species consortia models give information on bacteria adhesion, accumulation, and competition. The simplified combination of cariogenic bacteria reduces the complexity and difficulty of measuring ecological phenomena. It produces metabolic data of the chosen species under an interactional circumstance. Microcosm biofilms evolved from dental plaque or saliva were used in 11 studies. They closely mimic the physiological and microbiological properties of natural dental plaque. The complexity, biodiversity, and heterogeneity of the natural cariogenic biofilm are well preserved [15]. Nevertheless, the bacterial populations of microcosm biofilms vary from different hosts, times, and environments.

Most of the recent studies used biofilm closed system models (31/37, 84%). Multi-well cell culture plates were used to incubate the cariogenic bacteria together with the enamel or dentin substrate in most studies (30/31, 97%) [16]. In a closed system model, a finite culture medium is provided in a sealed container such as plates or tubes. The growth conditions will change considerably with the consumption of the nutrients and the accumulation of metabolic products. Hence, the physiological and biological properties of the biofilm are not comparable with the natural ones. Closed system models were simple, repeatable, controllable, and inexpensive. A small number of the studies (6/37, 16%) used open system models such as the oral biofilm reactor [17,18,19] or artificial mouth model [20,21]. Open system models simulate the in vivo environment better than closed system models. They also allow for better regulation of biofilm growth rate and other variables. The continuous supply of nutrient medium and removal of waste metabolic products provide a steady state condition [22]. Nevertheless, the repeatability of the experimental result is low because of the heterogeneity of the biofilm in the open system. Besides, the possibility of contamination can be high due to the complexity of the construction.

### 3.2. Chemical Models

Among the 294 in vitro studies, most studies (257/294, 87%) used chemical models to generate artificial caries or demineralized lesions on enamel and/or dentin. Chemical models mimic the caries process through the use of acid or acid buffer to simulate demineralization and remineralization processes. When the acidity (pH) drops below a certain level (critical pH), saliva and plaque fluid cease to be saturated with calcium and phosphate. The enamel hydroxyapatite will dissolve and demineralization of enamel occurs. This is represented with a simplified chemical reaction: Ca_10_(PO_4_)_6_(OH)_2_ + H^+^ 󠆂↔ Ca^2+^ + HPO_4_^2−^ + H_2_O. The left to right direction is demineralization. When calcium (Ca^2+^), phosphate (PO_4_^3−^), and hydroxyl (OH^−^) ions are accumulated, demineralization slows down to the moment when the saliva reaches saturation. When the pH goes up, re-deposition of minerals (remineralization) will occur and the reaction shifts from right to left.

Chemical models simplify the complex biofilm metabolism [23]. They aim to reflect the environment of the oral cavity to a chemical level rather than biological level [23]. The advantages of chemical models include simplicity of a study, low cost, efficiency (time saving), reproducibility, and stability of the experiment. A main disadvantage of chemical models is that they completely ignore the microbiological aspect of the caries formation. 

## 4. Types of Chemical Models Used in Recent Studies 

In this literature search, the chemical models used in recent studies could be further classified as simple mineralization models (159/257, 62%) and pH-cycling models (98/257, 38%). 

### 4.1. Simple Mineralization Models

Simple mineralization models use simple demineralization agents of low pH value. This method cuts down the remineralization process to create demineralized lesions. Mild organic acids and acid buffers such as lactic acid and acetate acid are used to create demineralized lesions. These mild acids would create demineralized lesions that are more similar to natural caries than inorganic acids. Substrates such as enamel block are immersed in acidic solution to create a demineralization zone on the surface. Normally, one single solution with a stable pH value will be used in the process of caries generation. The optimal pH value to create a subsurface lesion ranges from 4.4 to 5.0 in most experimental designs. Hydrochloric acid, phosphate acid, and citric acid are used to create acid erosion models. These inorganic acids are more acidic than those used for cariology research, and their pH values can be as low as 1.0.

Some studies used substrates topped with an acidic gel to generate caries-like lesions. The two common acidic gels used are carboxymethylcellulose-based lactic acid gel and ethylenediaminetetraacetic (EDTA) acid gel [12]. The demineralization process will stop after mineral saturation is reached. In this status, the mineral gain and loss of the substrates will be in a dynamic balance. The amount and viscosity of the gel applied on the top of the substrate will affect the time of mineral saturation. The contact area of the tooth tissue and demineralization gel also has an impact on mineral saturation. For the carboxymethylcellulose-based lactic acid gel, the consistency of the gel provides a diffusion barrier on the surface of the substrates. The diffusion process of the ions is slower than the demineralization solutions [24]. In addition, the carboxymethylcellulose may combine with calcium and reduce the demineralizing activity of calcium [25]. With the same exposure time as methylcellulose gel on the surface of substrates, the lesions created by EDTA gel can be very deep and nearly all the mineral is eradicated. EDTA gel might not simulate subsurface lesions [12]. 

Many researchers use simple mineralization models because they minimize the time and operational steps of the experiments. These artificially created demineralized lesions mimicking caries lesions and acid erosion lesions are regarded as acceptable by some researchers [12]. Some studies added in a thermos-cycling protocol into a mineralization model to simulate the aging process of the specimens studied [26,27]. The extent of demineralization can be regulated by a number of factors such as acidity, time, temperature, mineral concentration, and mineral dissolution inhibitors [28]. By modifying these factors, the characteristics of lesions such as lesion depth, mineral loss ratio, and gradient of mineral lost can be controlled [12]. The demineralized lesions induced using simple mineralization models show a higher mineral loss ratio than natural caries [12]. Moreover, the study designs and the acid used in most recent studies are not the same. The physical and mechanical properties of the artificially created carious lesions are different. Hence, the results of these studies are not comparable and the conclusions drawn could be different [24]. It is important to interpret the results of these studies with caution.

### 4.2. PH-Cycling Model

The pH-cycling model was invented and first published in 1982 by Cate and Duijsters [29]. The model is based on a scheme in which a pH neutral environment was periodically interrupted by acid challenges. It intends to mimic the in vivo periodic alternation of pH, much as it occurs in the mouth when sugars are metabolized, to form a caries lesion. These combination experiments are designed to mimic the dynamics of mineral loss and gain involved in caries formation, which is an important advantage of the pH-cycling model. Other advantages include the high level of scientific control and the resulting lower variability intrinsic to in vitro models, as well as the smaller sample size required. Additionally, the response variables that can be employed in pH-cycling models are more sensitive than those available for use in a clinical situation [30]. The remineralization solution is often neutral in acidity (pH = 7). The pH used for demineralization solution varied from 4.4 to 5.5, depending on different substrates and demineralized acid. The duration of pH cycling ranges from a few days to months. pH-cycling models were often used to study dentin caries [31]. Dentin caries is a biochemical process characterized initially by the dissolution of the mineral part, thus exposing the organic matrix to breakdown by bacteria-derived and host-derived enzymes [32]. Solutions simulating plaque fluid conditions could be used to reduce the difference between pH cycling models and in vivo situations [33].

## 5. Conclusion

In conclusion, in vitro studies have been the most common method adopted for cariology research in recent studies. The easily controlled parameters of the models enabled the forthright regulation of model sensitivity to adapt to varied requirements of testing. In vitro studies have the advantages of simplicity, low cost, and efficiency (time saving). However, they cannot mimic all the factors affecting the caries formation process. The most common model used in in vitro studies was a chemical model. The advantages of chemical models include simplicity of the study, low cost, efficiency (time saving), reproducibility, and stability of the experiment. However, the caries generated are not biological and chemical models, and cannot mimic a carious lesion in the complex oral environment. Researchers should interpret the results and conclusions of these studies with caution. No study and model could suit all experimental designs. Different models have their own strengths and limitations when used for mechanistic studies on demineralization-remineralization. The criteria of selecting a model should meet the requirements of the experimental objectives.

## References

[1] Selwitz R.H., Ismail A.I., Pitts N.B. (2007). Dental caries. Lancet.

[2] ten Cate J.M. (2015). Models and role models. Caries Res..

[3] Drake M.A. (2003). Encyclopedia of Library and Information Science.

[4] Falagas M.E., Pitsouni E.I., Malietzis G.A., Pappas G. (2008). Comparison of pubmed, scopus, web of science, and google scholar: Strengths and weaknesses. Faseb J..

[5] Sung Y.-H., Kim H.-Y., Son H.-H., Chang J. (2014). How to design in situ studies: An evaluation of experimental protocols. Restor. Dent. Endod..

[6] White D.J. (1992). The comparative sensitivity of intra-oral, in vitro, and animal models in the ‘profile’ evaluation of topical fluorides. J. Dent. Res..

[7] Klinge B., Jönsson J. (2011). Animal models in oral health sciences. Handbook of Laboratory Animal Science, Volume II, Third Edition: Animal Models.

[8] Bowen W.H. (2013). Rodent model in caries research. Odontology.

[9] Tang G., Yip H.K., Cutress T.W., Samaranayake L.P. (2003). Artificial mouth model systems and their contribution to caries research: A review. J. Dent..

[10] Bowen W.H. (2016). Dental caries—Not just holes in teeth! A perspective. Mol. Oral Microbiol..

[11] Salli K.M., Ouwehand A.C. (2015). The use of in vitro model systems to study dental biofilms associated with caries: A short review. J. Oral Microbiol..

[12] Schwendicke F., Eggers K., Meyer-Lueckel H., Dorfer C., Kovalev A., Gorb S., Paris S. (2015). In vitro induction of residual caries lesions in dentin: Comparative mineral loss and nano-hardness analysis. Caries Res..

[13] Bowden G. (1995). The role of microbiology in models of dental caries: Reaction paper. Adv. Dent. Res..

[14] Chu C.H., Mei L., Seneviratne C.J., Lo E.C.M. (2012). Effects of silver diamine fluoride on dentine carious lesions induced by streptococcus mutans and actinomyces naeslundii biofilms. Int. J. Paediatr. Dent..

[15] McBain A.J. (2009). Chapter 4: In vitro biofilm models: An overview. Adv. Appl. Microbiol..

[16] Mei M.L., Li Q.L., Chu C.H., Lo E.C.M., Samaranayake L.P. (2013). Antibacterial effects of silver diamine fluoride on multi-species cariogenic biofilm on caries. Ann. Clin. Microb. Anti..

[17] Horie K., Shimada Y., Matin K., Ikeda M., Sadr A., Sumi Y., Tagami J. (2016). Monitoring of cariogenic demineralization at the enamel-composite interface using swept-source optical coherence tomography. Dent. Mater..

[18] Zhou Y., Shimada Y., Matin K., Sadr A., Sumi Y., Tagami J. (2016). Assessment of bacterial demineralization around composite restorations using swept-source optical coherence tomography (SS-OCT). Dent. Mater..

[19] Tezuka H., Shimada Y., Matin K., Ikeda M., Sadr A., Sumi Y., Tagami J. (2016). Assessment of cervical demineralization induced by streptococcus mutans using swept-source optical coherence tomography. J. Med. Imaging.

[20] Schwendicke F., Diederich C., Paris S. (2016). Restoration gaps needed to exceed a threshold size to impede sealed lesion arrest in vitro. J. Dent..

[21] Kramer N., Mohwald M., Lucker S., Domann E., Zorzin J.I., Rosentritt M., Frankenberger R. (2015). Effect of microparticulate silver addition in dental adhesives on secondary caries in vitro. Clin. Oral Investig..

[22] Coenye T., Nelis H.J. (2010). In vitro and in vivo model systems to study microbial biofilm formation. J. Microbiol. Meth..

[23] Skucha-Nowak M., Gibas M., Tanasiewicz M., Twardawa H., Szklarski T. (2015). Natural and controlled demineralization for study purposes in minimally invasive dentistry. Adv. Clin. Exp. Med..

[24] Moron B.M., Comar L.P., Wiegand A., Buchalla W., Yu H., Buzalaf M.A.R., Magalhaes A.C. (2013). Different protocols to produce artificial dentine carious lesions in vitro and in situ: Hardness and mineral content correlation. Caries Res..

[25] Lynch R.J.M., ten Cate J.M. (2006). The effect of lesion characteristics at baseline on subsequent de- and remineralisation behaviour. Caries Res..

[26] Alsayed E.Z., Hariri I., Sadr A., Nakashima S., Bakhsh T.A., Shimada Y., Sumi Y., Tagami J. (2015). Optical coherence tomography for evaluation of enamel and protective coatings. Dent. Mater. J..

[27] Ozgul B.M., Tirali R.E., Cehreli S.B. (2016). Effect of biodentine on secondary caries formation: An in vitro study. Am. J. Dent..

[28] Marquezan M., Correa F.N.P., Sanabe M.E., Rodrigues L.E., Hebling J., Guedes-Pinto A.C., Mendes F.M. (2009). Artificial methods of dentine caries induction: A hardness and morphological comparative study. Arch. Oral Biol..

[29] ten Cate J.M., Duijsters P.P.E. (1982). Alternating demineralization and remineralization of artificial enamel lesions. Caries Res..

[30] Buzalaf M.A.R., Hannas A.R., Magalhaes A.C., Rios D., Honorio H.M., Delbem A.C.B. (2010). Ph-cycling models for in vitro evaluation of the efficacy of fluoridated dentifrices for caries control: Strengths and limitations. J. Appl. Oral Sci..

[31] Zhao I.S., Mei M.L., Li Q.L., Lo E.C., Chu C.H. (2017). Arresting simulated dentine caries with adjunctive application of silver nitrate solution and sodium fluoride varnish: An in vitro study. Int. Dent. J..

[32] Mei M.L., Lo E., Chu C. (2016). Clinical use of silver diamine fluoride in dental treatment. Compend Contin. Educ. Dent..

[33] Lynch R.J.M., Mony U., ten Cate J.M. (2007). Effect of lesion characteristics and mineralising solution type on enamel remineralisation in vitro. Caries Res..

